# CoSREM: a graph mining algorithm for the discovery of combinatorial splicing regulatory elements

**DOI:** 10.1186/s12859-015-0698-6

**Published:** 2015-09-04

**Authors:** Eman Badr, Lenwood S. Heath

**Affiliations:** Department of Computer Science, Virginia Tech, Blacksburg, Virginia, USA

**Keywords:** Algorithms, Graph mining, Alternative splicing, Splicing regulatory elements

## Abstract

**Background:**

Alternative splicing (AS) is a post-transcriptional regulatory mechanism for gene expression regulation. Splicing decisions are affected by the combinatorial behavior of different splicing factors that bind to multiple binding sites in exons and introns. These binding sites are called splicing regulatory elements (SREs). Here we develop CoSREM (Combinatorial SRE Miner), a graph mining algorithm to discover combinatorial SREs in human exons. Our model does not assume a fixed length of SREs and incorporates experimental evidence as well to increase accuracy. CoSREM is able to identify sets of SREs and is not limited to SRE pairs as are current approaches.

**Results:**

We identified 37 SRE sets that include both enhancer and silencer elements. We show that our results intersect with previous results, including some that are experimental. We also show that the SRE set *GGGAGG* and *GAGGAC* identified by CoSREM may play a role in exon skipping events in several tumor samples. We applied CoSREM to RNA-Seq data for multiple tissues to identify combinatorial SREs which may be responsible for exon inclusion or exclusion across tissues.

**Conclusion:**

The new algorithm can identify different combinations of splicing enhancers and silencers without assuming a predefined size or limiting the algorithm to find only pairs of SREs. Our approach opens new directions to study SREs and the roles that AS may play in diseases and tissue specificity.

**Electronic supplementary material:**

The online version of this article (doi:10.1186/s12859-015-0698-6) contains supplementary material, which is available to authorized users.

## Background

Alternative splicing (AS) is the primary explanation for the difference between the estimated 24,000 protein-coding genes in the human genome and the estimated 100,000 different proteins that are synthesized [1, 2]. AS is a post-transcriptional mechanism for regulating gene expression and generating proteomic diversity [3, 4]. In AS, genes generate different mRNA isoforms from the same primary transcript [5, 6]. Recent studies show that AS occurs in more than 95 % of human genes [4, 6, 7].

The RNA splicing process depends on recognition, by the spliceosome, of specific sequence elements in pre-mRNAs called core splicing signals. These include the 5’ splice site, the 3’ splice site, and the branch point sequence [8].

AS is regulated by specific proteins, called splicing factors. There are 71 known human splicing factors [9, 10]. Splicing factors, such as SR proteins and hnRNPs, bind to certain short subsequences on the pre-mRNA, called splicing regulatory elements (SREs). Identifying these SREs and their combinatorial effects are crucial to understanding AS. Exonic SREs are classified as exonic splicing enhancers (ESEs) if they promote exon inclusion and as exonic splicing silencers (ESSs) if they inhibit exon inclusion [4, 5, 11]. Accurate splicing is crucial, as it is believed that mutations either in the core splicing signals or in the SREs contribute to approximately 50 % of human genetic diseases [1, 6, 12].

There have been several large-scale studies of AS. Several techniques were utilized to identify SREs, such as systematic evolution of ligands by exponential enrichment (SELEX) [13], UV crosslinking and immunoprecipitation (CLIP) [14], and minigene-based systems [15].

Beside experimental approaches, various computational approaches were developed to identify SREs. The word count enrichment approach is one widely used technique [4, 16–18]. Another approach utilizes machine learning methods such as support vector machine classifiers [19], while regression based methods were utilized as well [2]. We [20] developed a de Bruijn graph based model to identify SREs of varying lengths.

Most of these approaches have focused on individual motifs [21]. However, many AS events involve multiple regulators. Zhang et al. [22] showed experimentally that knocking out (mutating) from two to four ESEs affects splicing efficiency dramatically. Hence, AS is a complex process that involves cooperative or competitive interplay between splicing enhancers and silencers. Most tissue-specific AS events studied so far seem to be regulated by a more complex group of regulators [8, 11].

For example, if an exon has both ESE and ESS elements in proximity and in case of having an SR splicing factor with great affinity (SR factors are proteins that bind to enhancers and play various roles in spliceosome assembly [8]), the SR protein will bind to the ESE and stimulate exon inclusion. This is through recruiting other spliceosome proteins, such as U1 and U2, to the core splicing signals. Consequently, the spliceosome machinery is assembled, and the exon is included.

On the other hand, if an inhibitory splicing factor such as hnRNP, which acts as a splicing repressor, is also present, it may inhibit the exon inclusion by binding to the silencer sequence and recruiting the binding of other inhibitory factors. These factors extend to the exon boundary and prohibit the binding of the SR protein. As a result, the exon will be skipped [8, 23].

In general, identifying individual *cis*-regulatory elements does not suffice to explain tissue-specific or condition-specific AS. The challenge is that, because of the large number of possible SRE pairs that reside in different regions, experimental approaches for identifying SRE pairs will be prohibitively expensive [24]. Identifying larger SRE combinations, where multiple SREs are working together, will be even harder.

Recent methods have studied combinatorial SREs in AS regulation [24, 25], but some of them did not exploit transcript expression data and focused only on frequently co-occurring SREs. All the methods concentrated on SRE pairs only [24–27].

Ke et al. [25] utilized a hyper-geometric test to discover sequence pairs that are over-represented in intronic regions flanking human exons. They identified more than 60,000 5-mer sequence pairs with a p-value ≤10^−4^. Friedman et al. [26] employed a similar approach except they utilized a Poisson approximation instead of a hyper-geometric test. They identified SRE pairs at the two ends of introns in both human and mouse. Wen et al. [24] developed a regression model based on biophysical principals for the regulation of AS. It captures both the main effects of individual SREs and the combinatorial effects of SRE pairs. The authors model the spliceosome assembling process with a simplified chemical reaction. The authors identified 196 6-mer sequence pairs from different tissues. Their model was limited to the interaction of at most two SREs.

We have developed CoSREM (Combinatorial SRE Miner), an algorithm for discovering combinatorial SREs. CoSREM is a two-level graph mining algorithm that we apply to our SRE graphs [20] to identify co-occurring sets of SREs. Our focus is on identifying sets of exonic splicing regulatory elements whether they are enhancers or silencers. Experimental evidence is incorporated through the SRE graphs to increase the accuracy of the results. The identified SREs do not have a predefined length, and the algorithm is not limited to identifying only SRE pairs as are current approaches. CoSREM is implemented as an open-source package (https://github.com/emanmostafabadr/CoSREM).

## Methods

### Preliminaries

We use terminology from formal language theory [28]. Let *Σ* be an alphabet, a finite set of symbols such as the DNA alphabet {*A*,*C*,*G*,*T*}. As defined in [20], for *k*≥1, the *k*-dimensional de Bruijn graph *G*=(*V*,*E*) over *Σ* is a directed graph with vertex set *V*=*Σ* ^*k*^, all length-*k* strings over *Σ*, and edge set

$$\begin{array}{@{}rcl@{}} E & = &\{(\sigma w, w\tau) \mid w\in\Sigma^{k-1}, \sigma,\tau\in\Sigma\}. \end{array} $$

In other words, an ordered pair of length-*k* strings (*u*,*v*) is in *E* if the length- (*k*−1) suffix of *u* equals the length- (*k*−1) prefix of *v* [29].

**Fig. 1:**
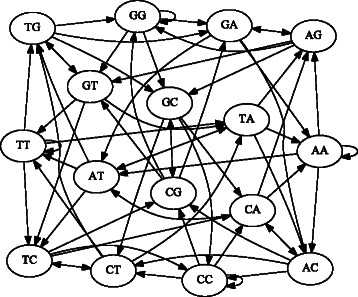
The 2-dimensional de Bruijn graph over the DNA alphabet {*A*,*C*,*G*,*T*}

For example, the 2-dimensional de Bruijn graph over the DNA alphabet *Σ*={*A*,*C*,*G*,*T*} has vertex set *V*={*A* *A*,*A* *C*,*A* *G*,*A* *T*,*C* *A*,*C* *C*,*C* *G*,*C* *T*,*G* *A*,*G* *C*,*G* *G*,*G* *T*,*T* *A*,*T* *C*,*T* *G*,*T* *T*}. See Fig. 1.

Let *G*=(*V*,*E*) be any de Bruijn graph, and let *U*⊆*V*. The *SRE graph G*_*U*_=(*U*,*E* ^′^ *) for G and U* is the vertex-induced subgraph of *G* with edge set

$$\begin{array}{@{}rcl@{}} E' & = &\{(u,v)\in E \mid u,v\in U\}. \end{array} $$

Let $G_{U_{\textit {ESE}}}$ be an SRE graph where the chosen vertex set *U*_*ESE*_ has experimental evidence of enhancing activity. In analogy to $G_{U_{\textit {ESE}}}$, let $G_{U_{\textit {ESS}}}$ be an SRE graph where the chosen vertex set *U*_*ESS*_ has experimental evidence of silencing activity. The SRE graph only includes 6-mers with the strongest experimental evidence among all the available 6-mers. It is the basic graph needed to extract SREs of different lengths and SRE sets as well.

Let *Y* be a set of *n* *k*-mers of interest *Y*={*y*_1_,*y*_2_,…,*y*_*n*_}. For example, it can contain only 6-mers with evidence of enhancing activity, in other words, 6-mers that correspond to the vertices in the $G_{U_{\textit {ESE}}}$ graph.

Let *X* be a set of *m* exons *X*={*x*_1_,*x*_2_,…,*x*_*m*_}. The *SRE profile matrix P*=(*p*_*i*,*j*_ *)* is the *n*×*m* occurrence matrix, where *p*_*i*,*j*_=1, if *k*-mer *y*_*i*_ is in exon *x*_*j*_, and *p*_*i*,*j*_=0, if *k*-mer *y*_*i*_ is not in exon *x*_*j*_. Let *P*_*ESE*_ and *P*_*ESS*_ be two SRE profile matrices for enhancers and silencers, respectively.

For a *k*-mer *y*_*i*_, *T*(*y*_*i*_) is the set of exons containing *y*_*i*_, that is, *T*(*y*_*i*_)={*x*_*j*_∣*p*_*i*,*j*_=1}.

Let *Y* ^′^⊆*Y* be a set of *k*-mers. The set of shared exons for *Y* ^′^ is $T(Y')=\bigcap _{y_{i} \in Y'} T(y_{i})$, the set of all common exons where the *Y* ^′^ *k*-mers reside together.

Let *G*_*S*_=(*S*,*E*_*S*_) be an induced connected subgraph of the SRE graph *G*_*U*_. *G*_*S*_ is *α*-cohesive if |*T*(*S*)|≥*α*, where *α*≥1. *G*_*S*_ is a *maximal α-cohesive subgraph (MCS)* if none of its supergraphs is *α*-cohesive. *MCSs* serve as the potential regulatory elements. They give us the ability to produce variable length SREs.

Let $M=\{G_{S_{1}},G_{S_{2}},...,G_{S_{r}}\}$ be a set of MCSs, where its shared exon set is $T(M)= \bigcap _{G_{S_{i}} \in M} T(S_{i})$. *M* is called an *MCS collection* if it satisfies the following conditions: |*M*|≥*β* and |*T*(*M*)|≥*θ*, where *β* and *θ* are user defined thresholds. An *MCS collection* is a set of SREs (enhancers or silences) that reside in the same set of exons.

### Problem definition

Let *C*={*M*_1_,*M*_2_,...,*M*_*l*_} be a set of all the MCS collections that can be identified given the two SRE graphs $G_{U_{\textit {ESE}}}$ and $G_{U_{\textit {ESS}}}$, SRE profile matrices *P*_*ESE*_ and *P*_*ESS*_, and the parameters *α*, *β*, and *θ*. The problem of discovering combinatorial SREs is to find the set *C* such that |*M*|≥*β*, |*T*(*M*)|≥*θ* for any *M*∈*C*, and |*T*(*S*)|≥*α* for any *G*_*S*_∈*M*. Specifically, the goal is to discover all SRE sets whose SREs co-occur in the same exons.

### Data sets

We utilize LEIsc (Log of the Enrichment Index, scaled) scores from [30]. The authors used a minigene approach to place all 4096 6-mers at five different sites in two model exons, which were then sequenced using an Illumina Genome Analyzer. They then transfected their library of minigenes into human embryonic kidney cells (HEK293) and, after 24 hours, isolated the mRNA molecules that included the central exon, converted these to cDNA, and sequenced the resulting DNA. An enrichment index was calculated based on the output proportion with respect to the input proportion. The enrichment index score represents the splicing efficiency of the central exon, with higher values representing greater exon inclusion. Using a *t*-test to compare each LEIsc value of a specific 6-mer with the average of the LEIsc values of molecules that do not contain this 6-mer, Ke et al. [30] identified 1182 potential ESEs and 1090 potential ESSs.

We also utilized all unique coding exons for known human genes available from the ENCODE project [31]. It includes 205,163 exons from 29,179 genes. Data was acquired from the RefSeq Genes track. The December, 2013, human genome assembly (GRCh38/hg38) was used.

For comparing our results with previously published results, several databases are utilized. SpliceAid-F [9] is a recent comprehensive database that includes all the experimentally verified splicing factors and their binding sites. It contains 71 splicing factors and 655 binding sites for human. We also used AEdb [32], which is a database for alternative exons and their properties. It is the manually curated component of the Alternative Splicing Database (ASD). The exon data in AEdb have been experimentally verified. We also utilized PESE and PESS data sets from [33] which contains 2096, and 1091 8-mers as enhancer and silencer elements, respectively.

### Overview of the computational method

A de Bruijn graph based model is developed, and a two-level graph mining algorithm is applied to discover enhancers and silencers that occur in the same set of exons. Experimental evidence that a specific *k*-mer has enhancing or silencing behavior is incorporated through the graph model. Our hypothesis is that combinatorial SREs can be discovered by their co-occurrence behavior in the same set of exons and the experimental evidence of their enhancing or silencing activities.

Utilizing a de Bruijn graph allows us to detect potential SREs of different lengths based on the experimental data from Ke et al. [30]. For example, if there are two 6-mers that overlap in five nucleotides and both of them have high LEIsc values, there is a greater probability that they form a potential 7-mer SRE. Suppose that the two 6-mers *GTCATC* and *TCATCC* have high LEIsc scores. Consequently, there is a good chance of having one 7-mer SRE with the sequence *GTCATCC*. The same applies with *m* consecutive 6-mers in the de Bruijn graph; if they all have high LEIsc values, then they can form one potential (*m*+5)-mer SRE [20].

Our model starts with constructing the 6-dimensional de Bruijn graph *G*=(*V*,*E*) over the DNA alphabet *Σ*={*A*,*C*,*G*,*T*} and associates each vertex with its rank based on LEIsc scores from Ke et al. [30]. The next step is **building the SRE graphs**. For example, if we are looking for ESEs, we select a subset *U*_*ESE*_⊂*V* that is associated with the highest LEIsc values. In the same manner, we select *U*_*ESS*_ to be the 6-mers with the lowest LEIsc values. As a result, we construct two SRE graphs, $G_{U_{\textit {ESE}}}$ for enhancers and $G_{U_{\textit {ESS}}}$ for silencers. The next step is **constructing the SRE profile matrices**, where we build profile matrices *P*_*ESE*_ and *P*_*ESS*_ for enhancers and silencers respectively. We apply the first level of the CoSREM algorithm (GenMCS) for **discovering maximal** ***α*** **-cohesive subgraphs (MCSs)**. Our goal in this level is to discover potential enhancer and silencer elements of different lengths where each element resides in a specific set of exons. With inputs $G_{U_{\textit {ESE}}}$ and *P*_*ESE*_, GenMCS generates several subgraphs, where each one represents a set of ESEs that resides in at least *α* exons. In addition, GenMCS is also applied with inputs $G_{U_{\textit {ESS}}}$ and *P*_*ESS*_ to discover potential silencers as well. Combining the output from the two runs of GenMCS, we then apply the second level of CoSREM for **identifying MCS collections**. MCS collections are sets of cohesive subgraphs, whether they represent enhancers or silencers, that occur in at least *θ* exons. The output is sets of potential regulatory elements that are grouped together. The final step is **filtering the resulted MCS collections**. Each subgraph in an MCS collection is mapped to the actual sequence in the associated exons. The resulting sequences are checked for overlapping. In case of overlapped sequences, they are replaced by one longer *k*-mer, which is evaluated to be included or eliminated in the final output.

### Building the SRE graphs

The 6-dimensional de Bruijn graph *G*=(*V*,*E*) over the DNA alphabet *Σ*={*A*,*C*,*G*,*T*} is constructed. The *G* graph has 4096 vertices and 16,384 edges. As mentioned earlier, we utilize the LEIsc scores (calculated in [30]) of potential exonic enhancers and silencers. If a specific 6-mer was found to be an enhancer or silencer, we use its associated LEIsc score. If it is defined as neutral, we consider its LEIsc value to be zero. We order all the scores in descending order and associate each vertex *v* in the *G* graph with its rank. The rank suggests the strength of the effect of a specific 6-mer on splicing. Hence, the higher the rank, the greater the evidence of the enhancing activity for that specific 6-mer, and the lower the rank, the greater the evidence of the silencing activity. Let *R* be a predefined number of ranks. A set *U*_*ESE*_ is constructed by choosing the top *R* vertices by rank to create the SRE graph $G_{U_{\textit {ESE}}}=(U_{\textit {ESE}},E')$, and the lowest *R* vertices by rank to create the SRE graph $G_{U_{\textit {ESS}}}=(U_{\textit {ESS}},E'')$ as well.

### Constructing the SRE profile matrices

Two SRE profile matrices (*P*_*ESE*_ and *P*_*ESS*_) are then constructed based on the vertices in the SRE graphs $G_{U_{\textit {ESE}}}$ and $G_{U_{\textit {ESS}}}$ respectively. Utilizing the human coding exon database, we set *p*_*i*,*j*_ equal to 1 or 0, according to the presence or absence of 6-mer *y*_*i*_ in exon *x*_*j*_. We limit the search for 6-mers in the exons to the first 50 nucleotides as we showed that extending the exonic flanking length does not affect the results significantly [20].

### Discovering maximal *α*-cohesive subgraphs (MCSs)

Given an SRE graph *G*_*U*_ and an SRE profile matrix *P*, the algorithm GenMCS from [34] is modified to find maximal *α*-cohesive subgraphs.

GenMCS takes as an input, in case of ESEs, the SRE graph ($G_{U_{\textit {ESE}}}$), the SRE profile matrix (*P*_*ESE*_), and the user-defined threshold *α*. It starts by pruning all vertices that do not satisfy the threshold requirement. Then, starting from each vertex as an initial subgraph G, GenMCS extends the subgraphs in a depth first search manner. Each initial subgraph G will be extended with its neighboring vertices. GenMCS checks if the extended subgraph *G* ^′^ with one neighbor vertex will generate an *α*-cohesive subgraph (i.e a subgraph with its vertices sharing at least *α* exons, where *T*(*G* ^′^)≥*α*). If this is the case, GenMCS will proceed in a depth-first fashion to extend *G* ^′^. If subgraph *G* cannot be extended without violating the *α* threshold, then *G* is a maximally *α*-cohesive subgraph. Two pruning strategies are applied in the original algorithm to reduce the search space: if the extended subgraph has been seen before or if it is subsumed by any of the other discovered cohesive subgraphs. We modified GenMCS not to apply the second pruning strategy as, due to the nature of our data, it is allowed to have overlapping subgraphs with common vertices as long as the common exons are not the same. These overlapped subgraphs represent different SREs with some common nucleotides.

**Fig. 2:**
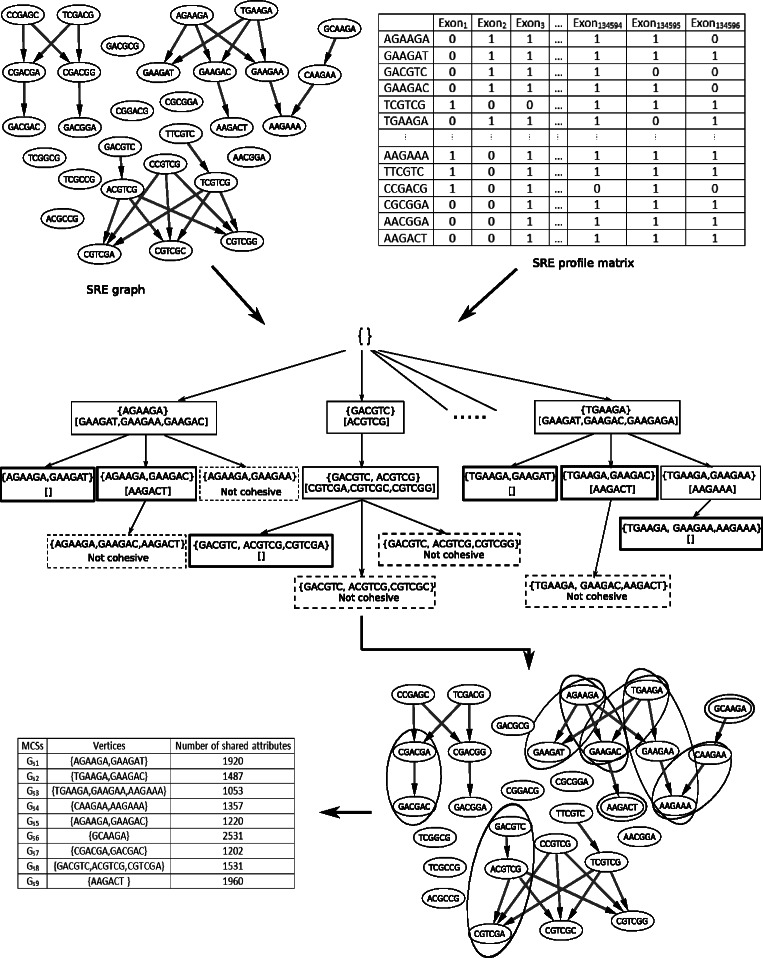
An example of mining cohesive subgraphs. The graph at the top left corner represents the SRE graph $G_{U_{\textit {ESE}}}$. We choose R = 30 which means the SRE graph contains the top 30 6-mers in rank. The matrix on the right is the SRE profile matrix *P*_*ESE*_. Setting *α*=1000 means that the connected vertices should co-occur in at least 1000 exons to be considered a cohesive subgraph. The tree in the middle shows how GenMCS proceeds. The bold boxes represent cohesive subgraphs. The dotted boxes represent subgraphs that are not cohesive and the remaining branch will be pruned. The output is 9 subgraphs as illustrated in the bottom graph

Figure 2 illustrates an example of the algorithm in case of ESEs. The output is a table called *MCStable*. It consists of maximal cohesive subgraphs and each subgraph is associated with a set of exons where the splicing enhancer, which this subgraph represents, resides. We apply GenMCS utilizing $G_{U_{\textit {ESS}}}$ and *P*_*ESS*_ as inputs to get potential silencers as well.

### Identifying MCS collections

The output from the first level of CoSREM is all the maximal *α*-cohesive subgraphs (MCSs), whether they represent enhancers or silencers, with their associated exons. The next step is to find collections of these already discovered subgraphs that share at least *θ* exons. To find such MCS collections, an *MCStree* is built.

The *MCStree* is a modified set enumeration tree, where each vertex contains an MCS collection *M* and its associated exons *T*(*M*). The root of the *MCStree* is a vertex with *M*=*∅* and *T*(*M*) containing all the exons.

**Fig. 3:**
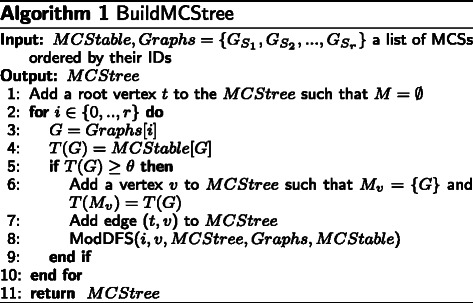
BuildMCStree algorithm: Build the *MCStree*

The algorithm, given in Fig. 3, uses a depth first search approach to build the *MCStree*.

**Fig. 4:**
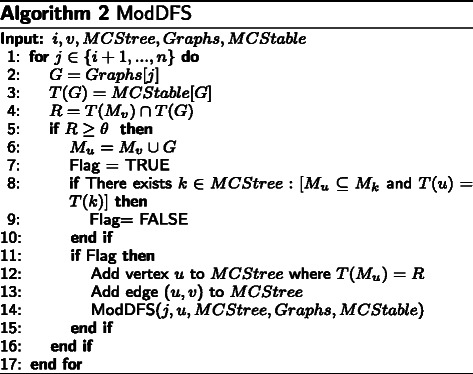
ModDFS: An algorithm to recursively extend the MCS collections

**Fig. 5:**
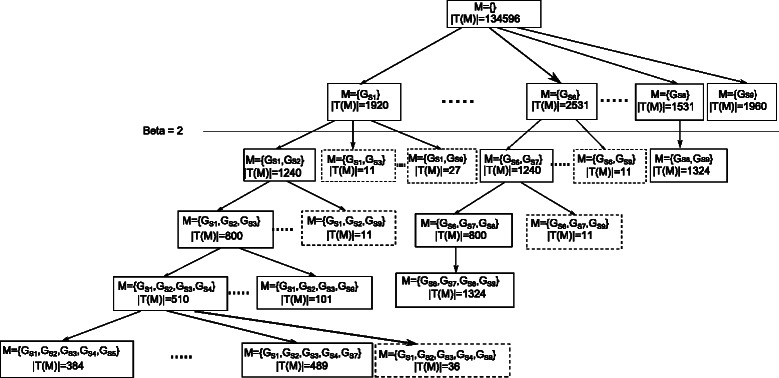
An example of an *MCStree*. The example shows a part of the tree where *θ*=100. The dotted boxes means that this MCS set does not satisfy the user threshold *T*(*M*)≥*θ*, where *T*(*M*) is the number of shared exons between the MCSs, and this branch will be pruned. all vertices with distance from the root ≥*β* threshold will be considered as potential MCS collection

It takes the *MCStable* as an input. *MCStable* is a hash table where the MCS IDs are the keys and the exon set of each MCS is the value. Each vertex at the first level of the tree represents one of the already calculated MCSs as an initial *M*. Therefore, the exon set *T*(*M*) is the exon set of the corresponding MCS (line 6). A child vertex *u* of vertex *v* is generated by extending *M*_*v*_ with one of the remaining MCSs and *T*(*M*_*u*_) is then calculated as depicted in Fig. 4. As we build the *MCStree* as an ordered tree, *M*_*v*_ is extended by adding an MCS whose ID is only bigger than the largest MCS ID in the collection. Different pruning strategies are applied to reduce the search time and space. However, they do not affect the accuracy of the produced results, as we prune only the branches that do not satisfy the user constraint. This follows from utilizing the set enumeration tree structure, where all set combinations are generated and tree branches are only pruned if constraints are violated. For example, one pruning strategy is that we extend the tree branches in a depth-first manner as long as the generated *M* in the current vertex has shared exons with size |*T*(*M*)|≥*θ*. Once this constraint is violated, this branch is pruned (see Fig. 4). This is analogous to the subset-infrequency pruning strategy utilized in Max-Miner algorithm [35]. Another strategy is to prune the branch if the generated *M* has been generated in a previous part of the tree with the same exon set. However, if the generated *M* is a subset of a previously generated MCS collection but with a different exon set, the new *M* will still be included. Figure 5 illustrates an example of an *MCStree*.

After building the *MCStree*, a breadth-first search (BFS) is applied to identify the path from the root to each vertex in the tree. Only vertices with distance ≥*β* from the root are included in the results. Each vertex represents an MCS collection and its distance from the root represents the number of MCSs in that collection (Fig. 5).

### Filtering the MCS collections

The output of CoSREM is all MCS collections, which represent sets of potential enhancers and silencers that co-occur in specific sets of exons. The goal of the filtering step is to generate the corresponding sequences for each MCS collection. As we allowed overlapping between sequences in the first level of CoSREM, there is a possibility to have multiple regulatory elements that form a co-occurring MCS collection but they are actually overlapping sequences in the exons. As a result, they can be considered as one longer *k*-mer instead. Therefore, we replace the overlapping SREs of the same type (ESEs or ESSs) with one longer SRE. That may result in an MCS collection with only one long SRE, or still multiple SREs if not all of them are overlapping. In the former case, this MCS collection will be eliminated from the results. On the other hand, if the set contains both enhancers and silencers, we allow overlapping between sequences as that is in accordance with the complex interplay between enhancers and silencers [8].

**Fig. 6:**
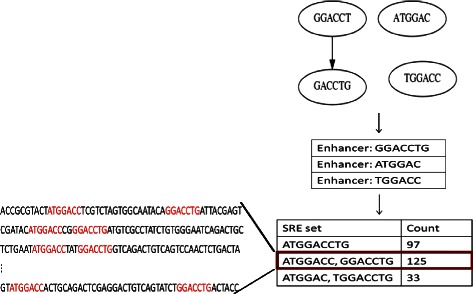
An example of generating SRE sets. The MCS collection here contains three subgraphs representing enhancers. Applying a modified depth first traversal will result in the longest sequence from each subgraph. The last step is to locate the three sequences in the associated exon set. If any of the sequences are overlapping in an exon, they will be merged in one longer sequence which results in new SRE sets. We then count the number of exons each new SRE set resides in. The SRE set that resides in at least 100 exons will be included in the final result as the set highlighted with a red rectangle

Therefore, for each MCS collection *M*, the corresponding sequences of each subgraph are generated. This is performed by applying a depth first traversal as in [20]. We eliminate the generated sequences that are subsumed by other sequences. Then, we check the first 50 nucelotides of each exon in the corresponding exon set *T*(*M*) to locate these sequences in the exon and generate a new SRE set if some of them are overlapping. For example, one of our MCS collections contains these four ESEs: *CCCGGA*, *CCGGAG*, *CGGAGC*, and *GGAGCC*. These sequences are found to overlap in some of the exons in the associated exon set, forming one 9-mer element *CCCGGAGCC*. In this case, we consider it only one ESE, and we do not include it in the final results. Another case was that only the first three ESEs overlap, forming an 8-mer sequence *CCCGGAGC*. This results in a new SRE set with two ESEs (*CCCGGAGC*, *GGAGCC*). It will be included in the final result if the number of exons, that this SRE set resides in, exceeds the original threshold for generating the MCS collection (*θ*≥100). Several other SRE sets are generated as well, based on the exons we are investigating such as (*CCCGGAG*, *CGGAGCC*), and (*CCCGGA*, *CCGGAGCC*). As a result, multiple SRE sets can be generated from one MCS collection, if they exceed the specified threshold. Figure 6 illustrates an example of the filtering process.

## Results

### Combinatorial SREs discovered in human exons

For predicting combinatorial SREs, we chose the highest and the lowest 400 6-mers by LEIsc values to construct the SRE graphs $G_{U_{\textit {ESE}}}$ and $G_{U_{\textit {ESS}}}$. These values were chosen since most of the analysis done by Ke et al. [30] on their produced LEIsc scores, which we utilize, was on the highest or the lowest 400 LEIsc scores. However, any number can be chosen based on the utilized data. We chose the user defined constraints *α*, *θ*, and *β* to be 1000, 100, and 2, respectively. Different values for *α* and *θ* have been tried as we will illustrate in the Discussion section. We chose *β* to be 2 to discover at least pairs of SREs.

GenMCS (the first level of CoSREM) produced 264 potential exonic regulatory elements as illustrated in Additional file 1: Table S1. That includes 175 enhancers and 89 silencers. Building the *MCStree* (the second level of CoSREM) generated 745 MCS collections as depicted in Additional file 1: Table S2. Filtering the results and generating the corresponding sequences, we generated 37 combinatorial SRE sets. That includes 30 sets of both enhancers and silencers and seven sets of co-occurring enhancers. The resulting regulatory elements lengths are between 6-mers and 7-mers. The results are shown in Tables 1 and 2 where we also utilized SpliceAid-F [9] and the ESEfinder tool [36] to evaluate the resulting regulatory elements and whether they bind to known splicing factors.

**Table 1:** Combinatorial enhancers and silencers generated by CoSREM. The number of exons each set resides in, and the splicing factors that they may bind to according to SpliceAid-F [9] and ESEfinder tool [36]

Combinatorial SREs	Number of exons	Splicing factors
CGGGAG,GGGAGG	526	hnRNP A1
GAAGGC,AGGCAG	373	9G8,SC35,SF2/ASF
GCTGTC,TGTCAG	254	-
GAGGAC,GGGAGG	233	SF2/ASF*, hnRNP A1
CCGGGA,GGGAGG	229	hnRNP A1
AGAGAC,TAGAGA	218	-
GGAGTC,AGTCAG	217	-
GAAGTC,AGTCAG	213	-
TGAGGA,GGTGAG	200	SF2/ASF
CCGGGAG,GGGAGG	199	hnRNP A1
GCGGGA,GGGAGG	190	hnRNP A1
GATGTC,TGTCAG	171	-
GCGGGAG,GGGAGG	169	hnRNP A1
AGAGGA,AGGCAG	156	FMRP
GCAAGA,GTGCAA	154	-
GTGAAGA,AGGTGA	153	SF2/ASF
GAGGAT,GGGAGG	147	SF2/ASF**,hnRNP A1
AGAGGA,CAGCCA	133	FMRP,hnRNP L
TGAGGA,AGGCAG	129	-
GATGCC,TGCCTA	127	SRp55*
GGAGCC,AGGTGG	114	-
GGAGCC,CCCACC	114	-
TGGACC,AGGTGG	112	-
TTCAAC,CTTTCA	112	SRp40*,hnRNP E1
TTCATC,CTTTCA	110	YB-1,SRp55*,hnRNP E1
GAACAA,AGGTGA	106	-
CAAGGA,CAGCCA	103	FMRP,hnRNP L
TGAGGA,AGGTGG	103	-
TGAGGA,AGGTGA	102	-
CAAGGA,TCCCAA	100	SRp40,FMRP

^*^identifies splicing factors identified by ESE finde

^**^means the splicing factors is identified by both methods

**Table 2:** Combinatorial enhancers generated by CoSREM. the number of exons each set resides in, and the splicing factors that they may bind to

Combinatorial SREs	Number of exons	Splicing factors
AGAGGA,TGAGGA	185	FMRP
GAAGGC,TGAGGA	113	9G8,SC35,SF2/ASF
CAAGGA,TGAGGA	105	-
AGAGGA,GATGGA	104	FMRP
AGAGGA,CAAGGA	103	FMRP
AGAGGA,GAGGAC	101	FMRP
GGAGCC,TGAGGA	100	-

Since our SREs are of variable length, as are SpliceAid-F binding sites, we checked if our elements is totally contained in at least one binding-site in the database or vice versa. Hence, we retrieved the associated splicing factor.

It should be noticed that although 37 combinatorial SRE sets were generated, the actual number of enhancer and silencer elements appeared in these sets are 25 and 14, respectively, with total number of 39 SREs. This supports the known complex relationship between enhancer and silencer elements and that alternative splicing is a complex process that involves cooperative or competitive interplay between both types. Our combinatorial SREs can be the basis to identify context-dependent regulation where the regulatory element behavior does not only depend on its sequence but also on its neighboring sequences [11].

**Fig. 7:**
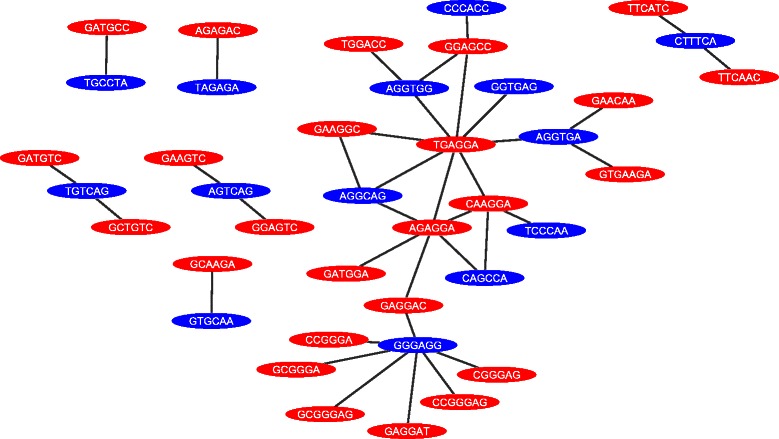
The regulatory network for enhancers and silencers. The red nodes represent enhancer elements, and the blue ones represent silencer elements. The network illustrates the many-to-many relationship between the enhancers and silencers

Figure 7 illustrates the the relationship between enhancer and silencer elements in our combinatorial SRE sets. It indicates the many-to-many relationship where, one enhancer element can co-occur with multiple silencers and vice versa. This many-to-many relationship does not only include regulatory elements of different types, it can also contains regulatory elements of the same type. For example the enhancer element *AGAGGA* co-occur with other enhancers (*C* *A* *A* *G* *A* *A*,*G* *A* *T* *G* *G* *A*,*T* *G* *A* *G* *G* *A*,*G* *A* *G* *G* *A* *C*).

### Comparison with other data sets

Among the 39 SREs in our combinatorial SRE sets, 35 were included in our previous results [20]. We also compared our results with previously published databases. We utilized exonic binding sites from SpliceAid-F [9]. SpliceAid-F contains 330 different sequences for human, 112 are exonic binding sites. We removed sites that bind to members of the extended family of heterogeneous nuclear ribonucleoproteins (hnRNPs) and other splicing factors that are considered silencers according to the literature. The remaining 59 sequences are considered as exonic enhancers, as they bind to splicing factors that are involved in enhancing activities. As stated earlier, since our predicted SRE list are of variable length, as well as SpliceAid-F binding sites, the overlap between the two sets are calculated by finding whether each sequence in the first list is totally contained in the second list or vice versa. Another database is AEdb [32]. It contains 294 splicing regulatory motifs. We only utilized human enhancers (64 sequences) and silencers (24 sequences). We utilized PESE and PESS data sets as well [33]. Table 3 summarizes the overlapping results. Overall, 88 % of the enhancers and 64 % of the silencers we identified in our combinatorial SRE sets can be mapped to previous data sets.

**Table 3:** Number of overlapped enhancers and silencers from our combinatorial SRE sets with previously published data sets. The numbers between brackets are the number of enhancer and silencer elements in our SRE sets

	SpliceAid-F	AEdb	PESE	PESS	
Enhancers (25)	8	7	19	-	
Silencers (14)	4	3	-	4	

We also compared our results with results from [30]. Those authors identified 232 and 262 6-mers that could have potential positive or negative synergy with other 6-mers. The authors did not identify an actual set of combinatorial 6-mers. From our 37 combinatorial SREs, 20 sets had at least one 6-mers from their list [30]. Most of the current approaches are applied on intronic regions [21, 24–27]. Therefore, we were not able to utilize their results for verification.

We also wanted to verify whether the SRE groups we found are significant. To address this issue, we randomly generated the same number of exons that we have in our database. Sequences of length 50 nucleotides that only include the letters *A*, *C*, *G*, and *T* were randomly produced. Then, we applied CoSREM with the same threshold values (*α*=1000, *θ*=100, and *β*=2). Although the number of generated MCS collections were considerably larger in the random case (4853), the filtering stage did not yield any results, as the generated groups did not pass the threshold *θ*=100. In other words, in the artificial data, the discovered SREs did not co-occur, although the specified threshold is relatively small. That means CoSREM is capable of distinguishing significant SREs that do not co-occur due to randomness in the data.

### SRE set (GAGGAC,GGGAGG) and the role it may play in cancer progression

We further investigated some of the combinatorial SREs. We chose the SRE set (*GAGGAC*, *GGGAGG*) as it is one of the highest ranked sets, according to the number of exons it resides in, and they are potential binding sites to both types of splicing factors (SR proteins and hnRNP proteins) as illustrated in Table 1. For example, when we checked the exons that the SRE set (*GAGGAC*, *GGGAGG*) resides in, the two SREs were overlapped in most of the sequences, constituting the sequence *GGGAGGACA*. We utilized the Human Splicing Finder tool [37] to validate whether the sequence contains both an enhancer and a silencer as we predicted. Human Splicing Finder is a tool to identify splicing motifs utilizing all the already known SRE experimentally and computationally. It also provides the splicing factors the sequence binds to if they are known. Utilizing Human Splicing Finder, the sequence *GGGAGGACA* is found to have the ESE motif *GGGAGGA*, among other motifs, where the splicing factor SF2/ASF binds. It also contains the ESS motif *GAGGAC* that binds to the splicing factor hnRNP A1.

**Fig. 8:**
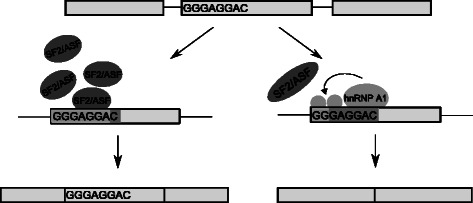
Possible combinatorial effect of the overlapped SREs (*G* *G* *G* *A* *G* *G* *A*,*G* *A* *G* *G* *A* *C*). One possible scenario is having SF2/ASF splicing factor with great affinity. It binds to the ESE and stimulate exon inclusion. Another possibility is if the splicing repressor hnRNP A1 exists, it may inhibit the exon inclusion by binding to the silencer sequence and recruit the binding of other inhibitory factors which extend to the exon boundary and prohibit the binding of the SF2/ASF protein. As a result, the exon will be skipped. The rectangles in this figure represent exons and lines represent introns

This is one of the known classical examples of the combinatorial effect of having both an ESE and an ESS in adjacent positions. There are several studies that report the antagonistic behavior between the SF2/ASF and hnRNP A1 splicing factors [8, 23]. For example, in exon 3 of the HIV1 tat gene, the hnRNP A1 splicing factor may bind to an ESS and inhibit splicing by propagating hnRNP A1 molecules further towards the 3’ splicing site. That propagation behavior can be inhibited by the SF2/ASF splicing factor when it binds to an ESE that resides upstream of the ESS, as in our sequence [8, 23, 38–40]. Furthermore, Mayeda et al. [41] showed *in vitro* that having different ratios of SF2/ASF to hnRNP A1 promotes exon skipping or inclusion by binding to different ESEs or ESSs. Therefore, that could provide us with an understanding of what might be the possible outcomes of combinatorial splicing regulation (Fig. 8).

**Fig. 9:**
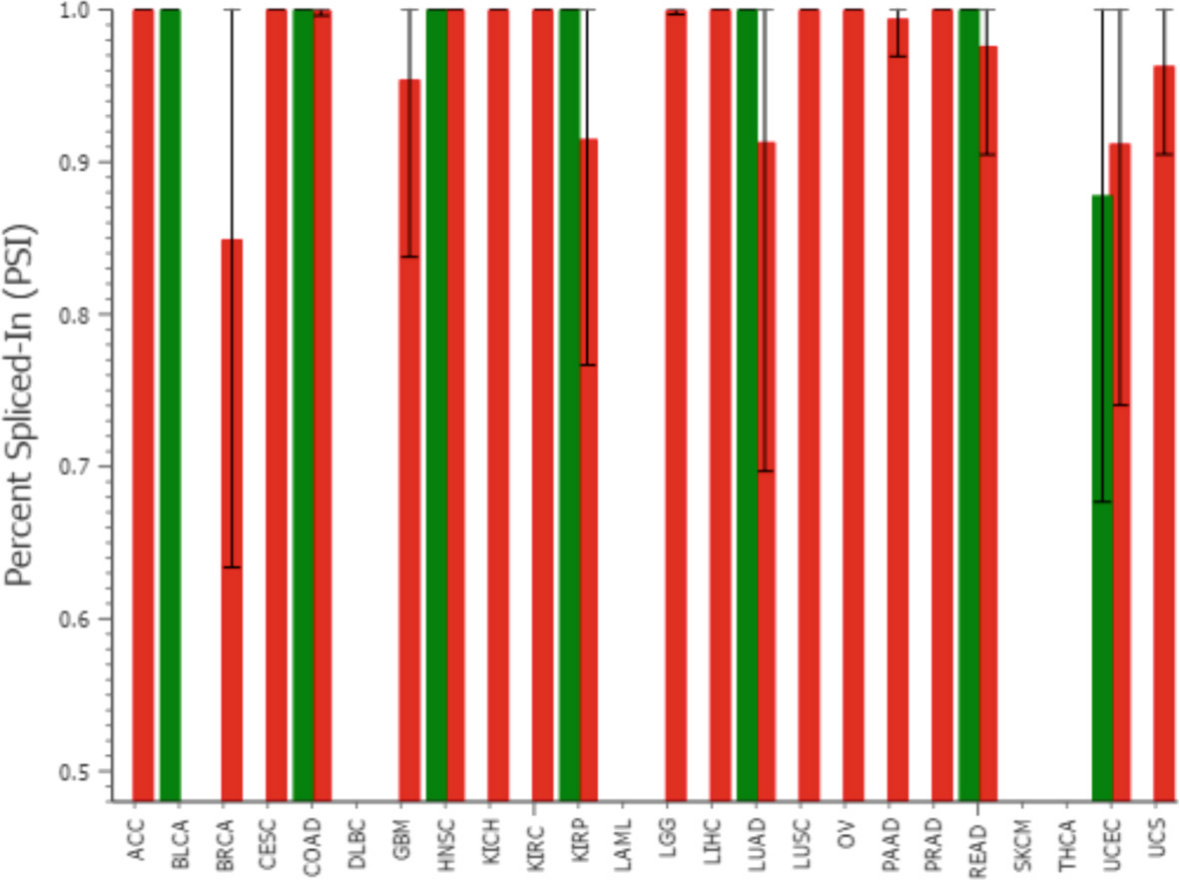
A bar plot of the PSI (Percent Spliced-In) values of exon 17 in PRKCG gene. It illustrates the difference in the PSI values between normal and tumor samples. The red bars represent the PSI of tumor samples while the green bars represent the normal samples. This figure is generated using TCGA Spliceseq [42]

We further investigated the exons in the genes that have this SRE set and identified by CoSREM utilizing TCGA Spliceseq [42]. TCGA is an AS database that utilizes RNA-Seq samples from The Cancer Genome Atlas project to provide the splicing patterns differences between different tumor samples and between tumor and normal samples. Several of these exons were found to be included in several samples of different cancer types and skipped in the normal samples. For example, exon 17 in the PRKCG gene is included in 100 % of all the transcripts of the samples for lung squamous cell carcinoma (LUSC), kidney renal clear cell carcinoma (KIRC), liver hepatocellular carcinoma (LIHC), prostate adenocarcinoma (PRAD), and kidney chromophobe (KICH), while skipped in 100 % of all the transcripts of the normal samples, as shown in Fig. 9. The inclusion or exclusion of these exons may be related to the antagonistic behavior of their positive and negative regulators that we identify. PRKCG is known to be a major receptor for phorbol esters, a class of tumor promoters. As abnormal splicing events are a major contributor to cancer development [43], understanding the reasons behind specific exon inclusion or exclusion can play a role in understanding cancer. The complete list of exon skipping events is shown in Additional file 1: Table S3.

We also utilized Ontologizer [44] to identify the enriched GO terms for the same set of genes. GO enrichment analysis is performed utilizing the Topology-Elim algorithm. Then, the Westfall-Young Single Step multiple testing correction procedure is applied. The most enriched biological process was "activation of Ras GTPase activity" with adjusted *p*-value 0.00028, meaning any process that initiates the activity of Ras superfamily members. It is known that Ras family genes are oncogenes [45–47]. Several human tumors have constitutively active Ras proteins. The activation can be caused by mutations in the Ras genes or by modifications in the upstream or downstream signaling components in Ras pathways [47]. Additional file 1: Table S4 contains the complete list of the biological processes that the predicted SRE sets are involved in.

### Tissue-specific combinatorial SREs

We performed a genome-wide analysis to study alternative splicing on multiple tissues (brain, heart, liver, and muscle) (Badr E., ElHefnawi M., and Heath L. S.: Computational identification of tissue-specific splicing regulatory elements in human genes from RNA-Seq data, submitted, 2015). The RNA-Seq data set from the Human BodyMap project [48] was utilized. We used DEXSeq [49] to identify tissue-specific exons. Then, we applied CoSREM, to identify combinatorial regulatory elements that may be responsible for exon inclusion or exclusion across tissues. Table 4 illustrates the number of discovered combinatorial SRE sets for each tissue.

**Table 4:** Number of exons used in CoSREM and the resulted combinatorial SREs. Taken from (Badr E., ElHefnawi M., and Heath L. S.: Computational identification of tissue-specific splicing regulatory elements in human genes from RNA-Seq data, submitted, 2015)

	No. of exons	Combinatorial SREs
Brain	8858	366
Heart	7818	283
Liver	4564	51
Muscle	2410	45

For each tissue, we identified a complicated regulatory network of enhancers and silencers with many-to-many relationship as stated earlier. We also identified two splicing factor proteins (FMRP, and HNRNPLL) that may have an antagonistic behavior that results in some exons being included in the brain tissue and excluded in the muscle tissue (Badr E., ElHefnawi M., and Heath L. S.: Computational identification of tissue-specific splicing regulatory elements in human genes from RNA-Seq data, submitted, 2015).

### Running time

Performance analysis of CoSREM is depicted in Fig. 11 with the actual numbers of generated MCSs and MCS collections are illustrated in Table 5. The first level of CoSREM, GenMCS, running time mainly depends on the number of discovered patterns as well as the number of explored branches [34]. For building the *MCSTree*, the time complexity is *O*(2^*r*^) in the worst case, where *r* is the number of MCSs. However, as stated before, multiple pruning strategies are used to reduce the time taken to build the tree. Traversing the tree utilizing the classic BFS algorithm takes *O*|*V*| where *V* is the number of nodes in the *MCStree*.

**Table 5:** CoSREM performance analysis for different values of *α*and *θ*, where time is in minutes

		*θ*=100		*θ*=300	
*α*	MCSs	MCS collections	Time	MCS collections	Time
50	1606	1	12.359	0	10.866
100	1232	1	14.576	0	8.181
150	1011	132	17.477	1	10.536
200	873	323	21.440	1	15.514
250	811	574	28.159	2	22.508
300	763	763	35.358	2	30.424
350	729	854	39.584	22	33.815
400	696	964	44.508	47	37.839
450	652	994	45.621	72	38.402
500	611	948	45.055	83	37.991
550	572	923	44.303	104	37.733
600	533	879	43.529	117	37.609
650	484	837	42.215	135	36.328
700	440	779	37.489	145	33.824
750	410	790	37.162	165	33.692
800	373	726	33.875	162	30.949
850	329	666	30.106	162	28.303
900	305	719	30.38	173	28.233
950	286	750	29.966	183	27.774
1000	264	745	28.797	186	26.782

## Discussion

We introduce CoSREM, a graph mining algorithm, to discover co-occurring groups of exonic enhancers and silencers. CoSREM utilizes experimental data to increase the accuracy of the results. Using a de Bruijn graph formalism allowed us to identify regulatory elements with different lengths without any prior assumptions on SRE size.

One of the advantages of our algorithm is its generality. CoSREM is designed to discover multiple SREs not only pairs as with the current approaches. Our current results do not include multiple SREs but the reason for that is the filtering step. In fact, the MCS collections that resulted from CoSREM include several larger groups of SREs, not only pairs (see Additional file 1: Table S2). As stated before, in the filtering step, we assume if SREs of the same type are overlapped, they constitute one longer SRE. This is one possibility to consider. Another possibility is that they are different regulatory elements that overlap and may have either cooperative or competitive behavior [50]. We chose to focus on the first possibility in our analysis. However, CoSREM provides the results for both possibilities. We provide both outputs in our open source package. So, the user can analyze both possibilities.

Another advantage is its flexibility. Utilizing a de Bruijn graph-based model allows building the main graph from any k-mer (based on the available data). The number of vertices chosen to build the SRE graphs can change according to the data as well. In our case, we utilized the LEIsc scores as a measurement for ranking 6-mers. The rank can be based on other criteria such as conservation scores or other data sources. For example, utilizing data from CLIP experiments where both the RNA binding protein and the location of its binding site is experimentally identified [14]. Having a list of all protein binding sequences that are experimentally verified can increase the probability of having a certain k-mer as a putative SRE if a part of the sequence is in that list. CoSREM can be applied on different parts of the genome as well to identify combinatorial SREs. For example, it can be applied to identify combinatorial SREs in both of the exonic flanking regions. It can be applied on intronic regions as well, depending on the data provided. We applied CoSREM on the first and last 50 nucleotides in the exons to discover SREs group that co-occur in both regions, and we found several co-occurring ESEs and, in some cases, the same ESE is repeated in these two different parts of the exon as shown in Additional file 1: Table S5.

**Fig. 10:**
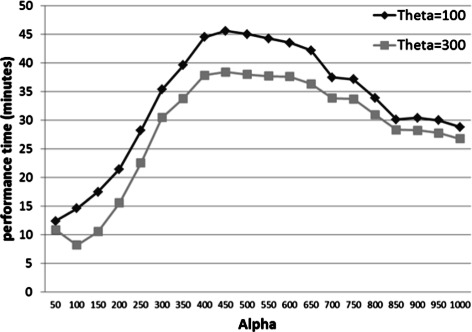
The number of generated MCSs and MCS sets using different values of *α* and *θ*

**Fig. 11:**
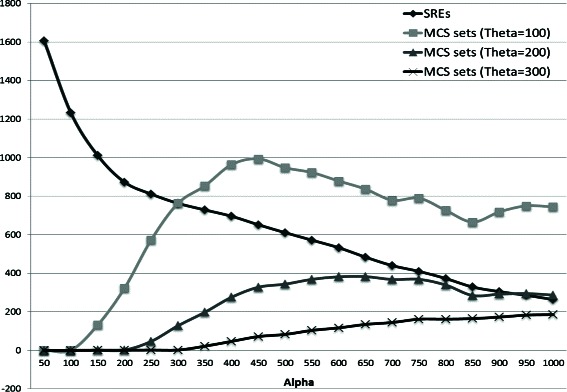
CoSREM time performance using different values for *α* in case of *θ*=100 and 300

Another aspect of CoSREM flexibility is the ability to choose the user defined thresholds. We have tried several values for the thresholds *α* and *θ*. As illustrated in Fig. 10, as *α* increases, the number of potential SREs decreases while the number of MCS collections increases and then decreases. This behavior can be explained, as *α* is the minimum number of exons that an SRE should reside in, and with increasing *α*, SREs that satisfy this constraint decreases and longer *k*-mer SREs are eliminated. However, as we set the *θ* threshold to a relatively small number (*θ*=100), some of these longer *k*-mers are combined again as co-occurring groups and this is the reason for the increasing number of combinatorial SREs. Eventually with the constant decreasing number of the resulted SREs, the number of the resulting MCS collections are decreased. We chose *α* to be 1000 to have a reasonable number of common exons between 6-mers to start with. Another reason is the time performance as shown in Fig. 11. The *θ* threshold eliminates only the groups with smaller exon sets. This is why we chose *θ* to be a small number relatively to have all the results for further filtering. We tried CoSREM with *α*=500 which resulted in 11 combinatorial SRE groups. These groups were a subset of our previous results with *α*=1000.

The ability to identify genes with different splicing events between normal and tumor samples, as in the case of the PRKCG gene, may shed further light on the important role that SREs may play in cancer progression and open the door for further experimental validation. Wan [51] introduces a protocol to manipulate the AS of exon 15 of the HER2 gene. Utilizing splice switching oligonucleotide (SSO), the splice site or an exonic enhancer is targeted to induce exon 15 skipping. That results in down-regulating the expression of HER2 mRNA and protein expression in HER2-overexpressing breast cancer cell line SK-BR-3. In fact, PRKCG has analogous behavior to HER2 where exon 17 is included in 100 % of the transcripts in case of LUSC, KIRC, LIHC, PRAD, KICH cancer samples and skipped in the normal tissues as we discussed earlier. That may open the way for further experimental validation.

## Conclusions

We have presented CoSREM, a graph mining algorithm to discover combinatorial SREs. Utilizing this approach allowed us to identify different combinations of splicing enhancers and silencers without assuming a predefined size or limiting the algorithm to find only pairs of SREs. Our approach can open new directions to study SREs and the roles that AS may play in diseases.

## Additional file

Additional file 1
**Supplementary Tables.** **Table S1.** The complete list of the biological processes that the predicted SREs are involved in. **Table S2.** MCSsets generated by CoSREM. **Table S3.** Exon skipping events identified for the SRE set (GAGGAC,GGGAGG). **Table S4.** The list of the biological processes that the predicted SREs are involved in. **Table S5.** Combinatorial SRE sets detected in both exonic flanking regions. (xlsx 73.7kb)
